# Zn, Cu, Cd and Hg binding to metallothioneins in harbour porpoises *Phocoena phocoena *from the southern North Sea

**DOI:** 10.1186/1472-6785-6-2

**Published:** 2006-02-07

**Authors:** Krishna Das, Arnaud De Groof, Thierry Jauniaux, Jean-Marie Bouquegneau

**Affiliations:** 1 MARE, Laboratory for Oceanology, B6c, Liège University, B-4000 Liège, Belgium; 2 Department of Pathology, Faculty of Veterinary Medicine, B43 Liège University, 4000 Liège, Belgium

## Abstract

**Background:**

Harbour porpoises *Phocoena phocoena *from the southern North Sea are known to display high levels of Zn and Hg in their tissues linked to their nutritional status (emaciation). The question arises regarding a potential role of metallothioneins (MTs) with regard to these high metal levels. In the present study, metallothionein detection and associated Zn, Cd, Cu and Hg concentrations were investigated in the liver and kidney of 14 harbour porpoises collected along the Belgian coast.

**Results:**

Metallothioneins seemed to play a key role in essential metal homeostasis, as they were shown to bind 50% of the total hepatic Zn and 36% of the total hepatic Cu concentrations. Renal MTs also participated in Cd detoxification, as they were shown to bind 56% of the total renal Cd. Hg was mainly found in the insoluble fraction of both liver and kidney. Concomitant increases in total Zn concentration and Zn bound to MTs were observed in the liver, whereas Zn concentration bound to high molecular weight proteins remained constant. Cu, Zn and Cd were accumulated preferentially in the MT fraction and their content in this fraction increased with the amount in the hepatocytosol.

**Conclusion:**

MTs have a key role in Zn and Cu homeostasis in harbour porpoises. We demonstrated that increasing hepatic Zn concentration led to an increase in Zn linked to MTs, suggesting that these small proteins take over the Zn overload linked to the poor body condition of debilitated harbour porpoises.

## Background

The harbour porpoise (*Phocoena phocoena*) is a small cetacean species inhabiting temperate and boreal waters [1]. The harbour porpoise is the most commonly seen porpoise, and is the most widely distributed of all cetaceans (whales and dolphins) in northern Europe. Because harbour porpoises live in coastal waters, they are affected by anthropogenic activities, such as accidental catches in fishing nets, depletion of prey species, noise and chemical pollution. Due to their top position in the trophic network, their long life span and their low rate of pollutant elimination, marine mammals, such as the harbour porpoise, can accumulate high levels of organochlorinated and metallic chemicals [2-4].

Recently, high levels of metals, such as Zn and Hg, have been measured in the liver and kidney of harbour porpoises from the North Sea [5-7]. Porpoises from the southern North Sea (northern France, Belgian and German coasts) were shown to have higher Zn and Hg concentrations compared to porpoises from Norwegian waters and the Baltic Sea, respectively [5,7]. These differences have been linked to both a difference in pollutant exposure and in nutritional status between individuals [5-7]. Elevated Zn concentrations in the liver have been shown to relate to degrading body condition [7]. Mean Zn concentrations ranged from 128 μg.g^-1 ^dw in non-emaciated porpoises to nearly 300 μg.g^-1 ^dw in emaciated porpoises from the southern North Sea [7].

In the liver and kidney, Zn, Cd and Cu are often bound to small cytosolic proteins, named metallothioneins [8]. Mammalian metallothioneins (MTs) are characterised by a low molecular mass of 6000 to 7000 Da, containing 60 to 68 amino acid residues, among which are 20 cysteines. MTs bind a total of 7 equivalents of bivalent metal ions [9,10].

The question arises regarding the potential involvement of metallothioneins in metal homeostasis during concomitant Zn increase and body condition degradation. In order to obtain a better understanding of the role of metallothioneins in the detoxification and the dynamics of Zn, Cu, Cd and Hg in the harbour porpoise, detection by gel chromatography and trace metal content of each obtained fraction were performed in the liver and kidney of 14 harbour porpoises collected along the Belgian coast.

## Results

### Metallothionein detection

**Figure 1:**
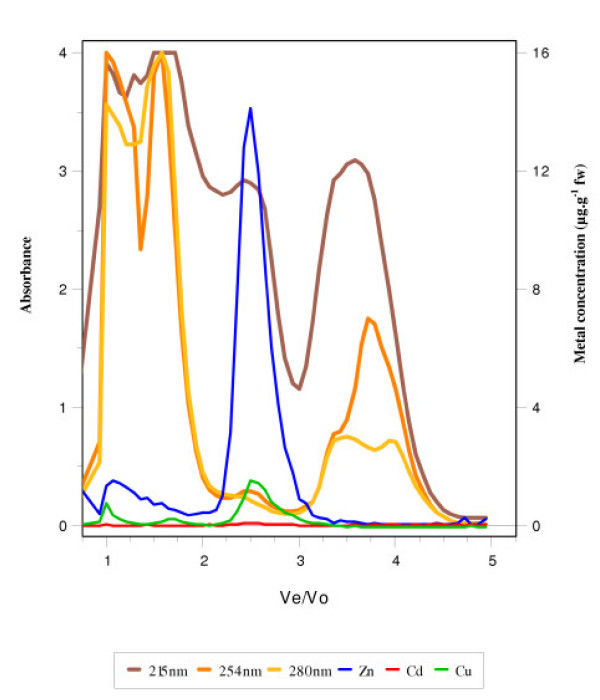
**Elution profile on AcA 54 column of the water soluble fraction extracted from the liver of one harbour porpoise *Phocoena phocoena (A00/568)***. Ve, elution volume; Vo, void volume of the column; mean volume per fraction, 2.46 ml Column height, 63 cm; diameter, 1.6 cm (Hg not detectable for this sample)

The typical elution profiles of metals for the harbour porpoise are shown in Figure 1. AcA-54 gel chromatography of the supernatant revealed a large band of metal (Zn, Cd, Cu) in the 10 kDa region of the profile, consistent with the presence of metallothionein-like proteins (MTs, fractions 33 to 43). This metal peak is associated with a high absorbance at 254 nm and low absorbance at 280 nm related to the lack of aromatic amino acids in these proteins. Peptide and Cd-cystein bonds are primarily responsible for the peaks at 215 and 254, respectively. The fractions eluted earlier and later than MTs were defined as HMWP (High Molecular Weight Proteins) and LMWP (Low molecular Weight Proteins), respectively.

### Zn, Cu, Cd and Hg distribution within the insoluble fraction and soluble proteins

Table 2 shows the metal (Zn, Cu, Cd, Hg) distribution in the insoluble and soluble fractions, including the metals bound to high molecular weight proteins, metallothionein-like proteins and smaller molecules. Whole tissue Zn, Cd, Cu and Hg concentrations in the liver, kidney and muscle have been presented elsewhere [7].

In the liver, 51% of the total Zn, 34% of the Cu and 48% of the total Cd were bound to cytosolic MTs, while Hg was mainly detected (more than 99%) in the insoluble fraction of the tissue. However, the percentages varied greatly, especially for Zn and Hg (Table 2).

**Figure 2:**
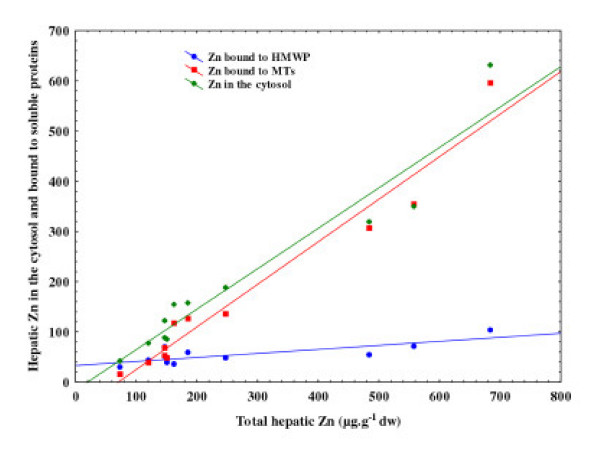
Relationship between the total hepatic Zn concentration and Zn concentration in the cytosol, bound to metallothioneins (MTs) and high molecular weight proteins (HMWPs).

**Figure 3:**
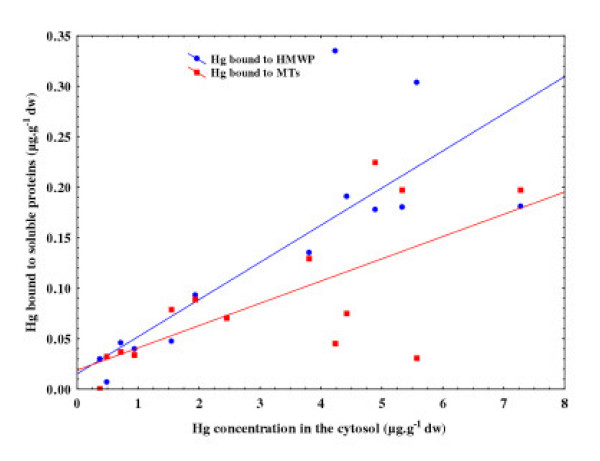
Relationship between Hg concentration in the cytosol and Hg concentration bound to metallothioneins (MTs) and high molecular weight proteins (HMWPs) in the liver.

An increase in total Zn concentration in the liver was followed by an increase in Zn concentration in the cytosol and Zn bound to cytosolic MTs (r = 0.8, p < 0.001), while Zn bound to high molecular weight proteins remained constant (Figure 2). By contrast, Hg content in the cytosol was correlated to both Hg bound to MTs (r = 0.7, p < 0.01) and to HMWP (r = 0.8, p < 0.001) (Figure 3).

In the kidney, Zn was found within the insoluble fraction (mean percentage 48%), bound to high molecular weight proteins (mean percentage 35%) and MTs (mean percentage 14%). 61% of the Cu concentrations were measured within the insoluble fraction, followed by MTs (mean percentage 20%) and HMWPs (mean percentage 19%). More than half of the total renal Cd was bound to MTs (mean percentage 56%), while Hg was mainly found within the pellet (mean percentage 76%).

## Discussion

In our previous study, high Zn and Hg concentrations were measured in the liver of harbour porpoises from the southern North Sea, compared with porpoises from Norwegian waters and the Baltic Sea, respectively [7]. Zn and Hg contents were assumed to be redistributed from muscle (and blubber) to the liver through the blood stream [7]. In the present study, we focused on the subcellular distribution of Zn, Cd, Cu and Hg in the liver and kidney of the harbour porpoise and the binding of these metals to metallothioneins.

### Subcellular distribution of Zn, Cd, Cu and Hg

More than half of the total Zn in the liver was found to be bound to MTs, whereas only 14% of the total Zn was linked to MTs in the kidney. Similar results have been previously observed for other marine mammal species, such as the Northern fur seal, the Dall's porpoise and the California sea lion [11], the sperm whale [12], the narwhal [13], the striped dolphin [14], the bottlenose dolphin [14] and the white-sided dolphin [15]. Zn has been shown to be essential to the structure and function of a large number of macromolecules and for over 300 enzymatic reactions [16]. Unlike Fe and myoglobin, there is no dedicated store for Zn and very little is known about its homeostasis [16,17]. It has been suggested that one function of MT is to serve as a store for Zn [16]. MTs are Zn acceptors because of the abundance of free sulphydryl groups. However the sulphydryl groups are highly reactive and Zn may be transferred from MTs to other proteins [18]. It is unclear why there is a higher percentage of Zn bound to MTs in the liver (51%) than in the kidney (14%). One hypothesis is that, during emaciation, Zn concentrations increase mainly in the liver but not in the kidney [7], which implies that Zn-thioneins are more often mobilized in the liver than in the kidney. Another hypothesis is that emaciated porpoises often have severe pulmonary or systemic infectious disease processes, and elevated hepatic Zn levels may reflect increased hepatic acute phase protein synthesis in response to these infectious/inflammatory processes [6]. In humans, it is well established that infection is associated with Zn redistribution, and in particular that concentrations in the liver rise as a result of acute-phase protein synthesis [19-21].

In the present study, Cu in the liver of the harbour porpoise was found to be mainly distributed either in the insoluble fraction (48%) or on metallothioneins (34%). Cu in the kidney was found preferentially in the pellet (61%) and on MT (20%). These results are in good agreement with previously described data for other mammal species. For example, Cu was solely (100%) detected in the pellet of the liver and kidney of bottlenose and striped dolphins from the Mediterranean Sea except for a few individuals in which Cu was associated with cytosolic MTs [14]. 30% and 42% of the total copper was found to be present in the pellet fraction of the Northern fur seal and the Dall's porpoise, respectively [11]. Cu is an essential cofactor for approximately a dozen cuproenzymes in which Cu is bound to specific amino acid residues in an active site [22]. Cu may also be highly toxic. In cells, due to its highly reactive nature, it would be extremely harmful for Cu(I) to exist as a free ion, where it could participate in reactions whose products ultimately damage cell membranes, proteins and nucleic acids [23]. The absence of any simple relationship between the hepatic concentration of Cu and its cytotoxic effects implies that intracellular distribution and speciation of the metal both influence its toxicity [24]. Cu is delivered to specific molecules by forming complexes with several cytosolic proteins, known as Cu chaperone proteins [22,23]. One possible Cu chaperone is MT, which may play a role in intracellular transfer and storage. Cu can induce MT synthesis, although Zn and Cd are the best known inducers [25]. Suzuki and co-authors [26] have suggested that MT plays an important role under these conditions as well, by acting as a Cu reserve. In black footed albatrosses and Dall's porpoises with low Cu levels (around 5 μg.g^-1 ^on a fresh weight basis), distribution of Cu in cytosol was shown to be relatively low (32 and 41%, respectively). In contrast, distribution of Cu in cytosol was shown to be relatively high (67%) in the liver of northern fur seals accumulating high levels of Cu (23 μg/g on a wet wt basis) [11].

As liver Cu concentrations increase, the metal often accumulates in granular form in lysosomes, the nucleus and other organelles [27]. Cu-MT accumulates in hepatic lysosomes of Cu loaded animals [26]. The lysosomal Cu is often relatively inert and its localisation appears to constitute part of the mechanism for its detoxification [28]. The change in hepatic Cu distribution during the progression of Wilson's Disease is consistent with this view, as the apparent transfer of Cu from the cytoplasmic to the lysosomal pool is associated with a reduction in its hepatoxic effects [28,29]. Little is known about the control of intracellular levels of Cu, but binding of Cu to MT has been suggested as a short-term response to increased intracellular concentration of this metal [28]. Cu sub-cellular distribution in the pellet and on MTs of the harbour porpoise reflects dynamic interactions between MT and the lysosomal pool in the liver and kidney.

Cd levels are known to remain quite low in the liver and kidney of the southern North Sea harbour porpoises, in comparison with porpoises from Icelandic coasts [7]. Cd was often near the detection limit in the liver of these harbour porpoises but, when it was detectable, it was bound to the MT fraction. In the kidney, Cd was either bound to the MT fraction (56%) or found in the pellet (33%). Marine mammals have been described as being able to tolerate high levels of Cd without showing renal damage [4,30], raising the question of detoxification pathways in these species. The role of MT in Cd detoxification in mammals has been often described [31], but more recently Cd-containing granules have been observed in the kidney of two white-sided dolphins [32]. These two individuals with high Cd concentrations exhibited electron dense mineral concretions of diameters of up to 300 nm in the basal membranes of the proximal tubule. Cd-containing granules could constitute a means of immobilisation and detoxification [32]. In striped and common dolphins from the Mediterranean Sea, the percentage of Cd bound to MTs were shown to be 27–69% in the liver, and 23–73% in the kidney [14]. In both organs, Cd was bound to MTs even when present at low concentrations in the tissues [14]. In the kidney of one white-side dolphin, found stranded on the Belgian coast, 75% of the Cd content was found to be in the cytosolic fraction bound to metallothioneins [15]. The fact that 33% of the renal Cd concentration could be found in the pellet suggests similar detoxification processes and dynamic interactions between MT and Cd granules found in the pellet for the harbour porpoise.

Very few harbour porpoises in our study had detectable Hg in the cytosolic fraction of their liver, it being nearly equally distributed on HMWP and MT fractions. In the kidney, 24% of the Hg could be measured in the cytosol, especially on the HMWP fraction.

Mercury in the liver was almost solely distributed in the insoluble fraction of the tissue, probably resulting from the well known formation of tiemannite (HgSe) in marine mammal livers and kidneys [33,34]. Toxic methyl mercury is believed to be taken up from the diet and transformed into inorganic Hg and then accumulated mainly in nuclear, lysosomal and mitochondrial fraction in the liver of northern fur seals [34].

### Dynamic of metal bound to metallothioneins

In marine mammals, Zn is generally correlated with Cu as a result of both antagonistic behaviour and binding to metallothioneins [32,35,36]. This positive relationship has been observed in the livers of by-catch porpoises from Norway but not for stranded porpoises from the southern North Sea, reflecting a severe homeostasis disturbance linked to both emaciation and high Zn concentrations [7]. Short-term fasting or prolonged starvation, through decomposition of energy storage and mobilisation of body reserves, is known to influence the metabolism of trace elements, such as Zn and Cu [37-41].

Das et al. showed that increased hepatic Zn concentrations were not linked to a loss of liver mass during emaciation [7], indicating that metal burdens also increased. In that study, increasing hepatic Zn concentration in harbour porpoises from the Belgian coast led to an increase in Zn linked to metallothioneins, but not in Zn linked to soluble high molecular weight proteins. This suggested that these low molecular weight metal-binding proteins involved in metal homeostasis and detoxification may take over the Zn overload resulting from proteolysis. In contrast, when Hg content increases in the cytosol, part of it can be found in the HMWP fraction and in the MT fractions.

## Conclusion

In the present study, MTs appeared to play a minor role in the binding and detoxification of Hg by the harbour porpoise. In contrast, MTs played a key role in Zn and Cu homeostasis in the harbour porpoise. For both Cd and Cu, dynamic interactions seemed to occur between the insoluble fraction (containing the lysosomes) and the MT pool. Increasing hepatic Zn concentration in the whole liver led to an increase in Zn linked to MTs, suggesting that these low molecular weight metal-binding proteins take over the Zn overload linked to emaciation. However the question arises as to whether emaciated porpoises can still afford the cost of metallothionein synthesis whilst consuming their protein and lipid reserves.

## Methods

### Sampling

14 fresh harbour porpoises found stranded along the Belgian coastline were sampled between December 1993 and May 2001. Post-mortem investigations were performed according to standard procedures detailed elsewhere [42]. Samples of liver and kidney were stored at -20°C until analysis.

### AcA 54 chromatography

3 to 4 grams of liver and kidney were homogenized using an Ultra-Turrax in a 0.01 Mol ammonium formiate (pH = 7.4) containing 10 mM sodium azide and 0.01% dithithreitol and were centrifuged at 26 000 g (60 min, 4°C). The supernatant was filtered at 4°C on an Ultrogel^® ^AcA 54 gel column (1.6 × 63 cm) at 4°C leading to a fractionation of cytosolic molecules. Ultrogel^® ^AcA is composed of a homogeneous network of polyacrylamide (5%) and agarose (4%) in bead form and was used according to manufacturer's recommendations. Linear fractionation ranges from 5000 to 70 000 daltons and the exclusion limit is 90 000 daltons. Fractions were collected and absorbance profiles read at 215, 254 and 280 nm.

### Trace metals analysis and MT concentration assessment

Zinc (Zn), cadmium (Cd), copper (Cu) and mercury (Hg) concentrations in the tissues were extracted in our study, as described in detail elsewhere [7]. Zn, Cd, Cu and Hg were also analysed in the different fractions resulting from centrifugation and gel chromatography. Metal concentrations are expressed as μg.g^-1 ^dry weight (dw). After adding nitric acid (65%) to tissue homogenate, supernatant, pellet (soluble and insoluble fraction resulting from centrifugation), and all the chromatography fractions, all were slowly heated to 100°C until completely digested. Samples were diluted using deionised water and filtered prior to heavy metal analysis (Zn, Cu and Cd) by atomic absorption spectrophotometry (ICPS: ARL 3510). Hg was analysed by flameless atomic absorption (Perkin-Elmer MAS-50A), as described previously [7]. Quality control measurements for total mercury included replicate analysis resulting in coefficients of variation <10% and analysis of certified material (DORM-1, NRC, Canada).

The Hg absolute detection limit was 10 ng corresponding to 0.13 μg.g^-1 ^fresh weight (fw) for an average of 1.5 g of sample analysed. Detection limits for Cu, Cd and Zn were, respectively, 0.18, 0.18 and 0.17 μg.g^-1 ^dw. Quality of the analyses was controlled through participation in an intercalibration programme ([43]; Table 1).

## Authors' contributions

KD conceived the study, analysed the data and drafted the manuscript. AD participated in data collection and data analysis. TJ performed necropsies and collected the samples. JMB coordinated the study and participated in its design and in the final revision. All authors read and approved the final manuscript.

## Figures and Tables

**Table 1:** Quality control results acquired during Quasimene intercalibration programme (values expressed in μg.g^-1 ^dw)

**QTM042BT **(muscle)	**Assigned value**	**Measured value**	**n**
Zn	38.75	39 ± 0.7	12
Cd	0.132	0.13 ± 0.02	12
Cu	1.82	1.8 ± 0.04	12
Hg	0.049	0.04 ± 0.003	6

**Table 2:** Distribution of Zn, Cu, Cd and total Hg in the insoluble fraction (centrifugation pellet), soluble fraction, high molecular weight proteins (HMWP), metallothionein-like proteins (MTs), and low molecular weight proteins (LMWP) of harbour porpoises stranded along the Belgian coast (concentration expressed in μg.g^-1 ^dw).

		**insoluble fraction**	**soluble fraction**		
			
			HMWP	MTs	LMWP
Liver	Zn n = 14	86 ± 58 (30 – 228)	53 ± 19 (29 – 104)	144 ± 165 (16 – 595)	1.8 ± 1.7 (<0.1–6.2)
		30 ± 20 %	19 ± 7 %	51 ± 58 %	~0%
	Cu n = 14	14 ± 12 (6 – 43)	5 ± 3 (1.3 – 12)	10 ± 11 (2 – 39)	<0.1 (<0.1 – 6.1)
		48 ± 41 %	18 ± 10 %	34 ± 38 %	~0%
	Cd n = 14	0.2 ± 0.2 (0.01 – 0.6)	0.5 ± 0.6 (<0.1 – 1.8)	1.1 ± 0.8 (0.1 – 27)	0.5 ± 1 (<0.1 – 3.4)
		9 ± 9 %	21.5 ± 26 %	48 ± 36 %	21.5 ± 43 %
	Hg n = 14	43 ± 51 (0.5 – 167)	0.1 ± 0.1 (<0.1 – 0.3)	<0.1 (<0.1 – 0.2)	<0.1(<0.1)
		99.5 ± 12 %	~0.25 ± 0.02%	~0.25%	~0%
Kidney	Zn n = 14	55 ± 12 (36 – 69)	40 ± 21 (23 – 105)	16 ± 5 (6 – 28)	4.4 ± 7 (<0.1 – 27)
		48 ± 10 %	35 ± 18 %	14 ± 4 %	<4 ± 6 %
	Cu n = 14	8.8 ± 2.2 (6 – 13)	2.8 ± 1.8 (0.4 – 6.1)	2.9 ± 1.6 (0.6 – 6)	<0.1 (<0.1)
		61 ± 15 %	19 ± 12 %	20 ± 11 %	~0%
	Cd n = 14	1.1 ± 1.8 (<0.1 – 5.6)	0.2 ± 0.6 (<0.1 – 0.9)	1.9 ± 2 (<0.1 – 5)	0.2 ± 0.8 (<0.1 – 1.7)
		33 ± 53 %	6 ± 18%	56 ± 59 %	5 ± 23 %
	Hg n = 10	3.2 ± 3.3 (0.7 – 12)	0.5 ± 0.3 (0.2 – 1.1)	0.3 ± 0.2 (0.1 – 0.4)	0.2 ± 0.1 (<0.1 – 0.5)
		76 ± 78%	12 ± 7%	7 ± 5%	5 ± 2%
